# Intergenerational transmission of violence in Bangladesh: Mediated through maternal attitudes towards intimate partner violence, disciplinary beliefs, and life satisfaction

**DOI:** 10.1371/journal.pone.0341887

**Published:** 2026-01-30

**Authors:** Ahmed Usama Fahim, Atika Aboni, Shirajoom Munira, Nishat Tasnim Toosty

**Affiliations:** Department of Statistics, University of Dhaka, Dhaka, Bangladesh; IFPRI: International Food Policy Research Institute, UNITED STATES OF AMERICA

## Abstract

**Introduction:**

Child discipline, while intended to instill appropriate behavior, often manifests as violent practices in low- and middle-income countries, including Bangladesh. Maternal exposure to violence, attitude towards intimate partner violence (IPV), and disciplinary beliefs serve as key determinants of physical disciplinary practices. These dynamics illustrate how exposure to violence in adulthood can shape parenting behaviors, highlighting the urgency of addressing cultural attitudes that sustain harsh physical discipline.

**Materials and methods:**

This study analyzed nationally representative cross-sectional data from the 2019 Bangladesh Multiple Indicator Cluster Survey (MICS) which included 30044 mother-child (children aged between 2 and 14 years) pairs. Physical disciplinary practice is analyzed as an ordered outcome, considering maternal experience of physical violence as the primary exposure along with their attitudes toward IPV and disciplinary beliefs as mediators. This study used ordinal logistic regression within a structural equation modeling framework and bootstrapping technique to analyze indirect associations, providing robust inference that accounts for sampling variability and accommodates binary mediators.

**Results:**

Mothers exposed to violence had significantly higher odds of physically disciplining their children (odds ratio, OR=1.77 and 95% confidence interval, CI=[1.60, 1.95]). Three mediators significantly increased the odds of adopting harsh physical disciplinary practice by 2% through maternal positive attitudes toward IPV, by 51% through their disciplinary beliefs, and by 6% through their overall life satisfaction. The total association indicated that maternal exposure to violence nearly tripled the odds (OR = 2.89 and 95% CI= [2.52, 3.31]) of physical disciplinary practices.

**Conclusion:**

This study suggested that supportive environment for children can be fostered by reducing violence against women, promoting mothers’ life satisfaction, and reshaping women’s perceptions of spousal abuse and disciplinary beliefs.

## 1. Introduction

### 1.1. Background and literature review

Child discipline is an indispensable constituent of raising children, which is designed to teach them acceptable conduct and self-control. Based on cross-country data, the global prevalence of corporal punishment ranges from 60–75%. In 58 countries where severity was disaggregated, approximately 17% of children experienced severe physical punishment in the past month, while about 43% experienced milder forms of physical punishment [1,2]. Evidence indicates that severe physical violence against children most often occurs in their own homes and is primarily administered by caregivers [3]. Despite often being justified as necessary for disciplining children, these measures are linked to developmental problems, strained parent-child relationships, greater child aggression, low self-esteem, and major depression, higher injury risk, etc. [1]. Consequently, international bodies, including the European Union (EU) and the United Nations (UN), advocate non-violent child rearing and legal bans on corporal punishment [4,5].

Violent child disciplinary practices are commonly socially accepted in low- and middle-income countries such as Bangladesh [6]. In parallel, intimate partner violence against mothers remains highly prevalent. Although distinct in forms and targets, these practices frequently co-occur within the same households affecting both women and children [7]. Previous studies found that the mothers who were exposed to IPV or possessed a positive attitude toward IPV were more likely to physically punish their children as a mean of disciplining them [8–11]. In fact, women’s lifetime exposure to violence shapes their disciplinary beliefs and parenting behavior, reinforcing norms that legitimate physical punishment [12,13]. These experiences and attitudes influence both the decision to use and intensity of physical punishment [9,13]. Despite these findings, most of the previous studies in Bangladesh, have reported connections between harsh disciplinary measures and negative outcomes for children, rather than directly linking these practices to mothers’ exposure to violence [6,13–15]. The intensity of children’s disciplinary practices being predominantly modeled as a binary outcome, has also been largely overlooked in the available research [6,15,16]. In particular, lower maternal satisfaction may increase the likelihood or intensity of harsh disciplinary practices, highlighting a potential pathway through which maternal well-being affects child outcomes [16]. To address these gaps, this study examines the severity of physical disciplinary practices using a composite index and evaluates the roles of maternal exposure to violence along with three different mediators.

Accordingly, this study aims to examine both the direct and indirect pathways linking maternal exposure to violence with child disciplinary practices, addressing the following research questions.

- Does mothers’ exposure to violence influence their use of physical disciplinary methods on children?
- Does mothers’ exposure to violence indirectly affect child physical discipline through their attitudes toward IPV, disciplinary beliefs, and life satisfaction?

The work also examined various socio-demographic indicators, both child and mother-level characteristics those may be associated with the child disciplinary practice. The objective of this study was to implement structural equation modeling (SEM) framework in order to find how the association between mothers’ exposure to violence and physical disciplinary practices is explained through the selected mediating factors. Such an approach is valuable for informing interventions designed to disrupt intergenerational cycles of violence by addressing how mothers’ reported experiences of violence in adulthood, within the scope of this study, may shape their attitudes and practices regarding child physical discipline [17].

### 1.2. Conceptual framework

**Fig 1:**
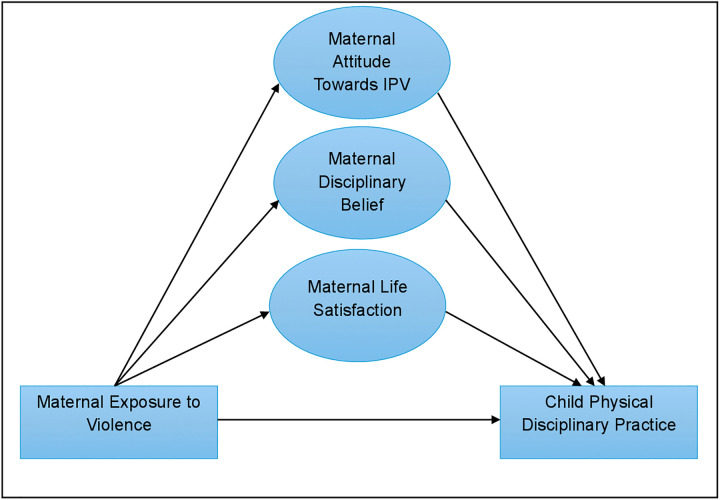
Conceptual framework examining pathways from maternal exposure to violence to the use of physical disciplinary practices.

This study explored how maternal exposure to violence influences physical disciplinary practices on children in Bangladesh. Exposure to violence is considered as the primary exogenous variable, as it heightens stress and normalizes aggression within families. The available research consistently [8,10,12,13] showed that mothers who support IPV are significantly more likely to physically punish their children. The mothers’ acceptance of IPV, their beliefs of endorsing physical punishment, and their life satisfaction, plausible correlates of the main exposure variable, were analyzed as mediators of the association between maternal exposure to violence and the use of physical disciplinary practices. The pathways among the target outcome, main exposure variable, and the mediators of interest are illustrated in **Fig 1**.

Although the data are cross-sectional, the temporal structure of the variables supports the assumed direction of pathways. Mothers’ exposure to physical attack was measured as a lifetime experience, while the mediators (attitudes toward partner violence, disciplinary beliefs, and life satisfaction) and the outcome (child disciplinary practice) reflect current states and behaviors. This provides a plausible temporal ordering in which past exposure precedes current mediators and parenting practices. These mediators are proximal, linking past maternal exposure to violence with later parenting practices, as attitudes and life satisfaction evolve through early socialization and adult experiences [18,19].

## 2. Materials and methods

### 2.1. Data source

The study utilized cross-sectional data from the 2019 Bangladesh Multiple Indicator Cluster Survey (MICS) conducted by the BBS in association with UNICEF Bangladesh. There were 3220 primary sampling units in this study. Two phases were used to sample the strata from urban and rural regions, yielding a total of 64400 households. MICS surveys employed computer-assisted personal interviews based on the CSPro (Census and Survey Processing System) software of version 6.3. The survey collected national level data on child discipline, protection from violence, and many socio-demographic characteristics concerning women and children.

### 2.2. Participants

**Fig 2:**
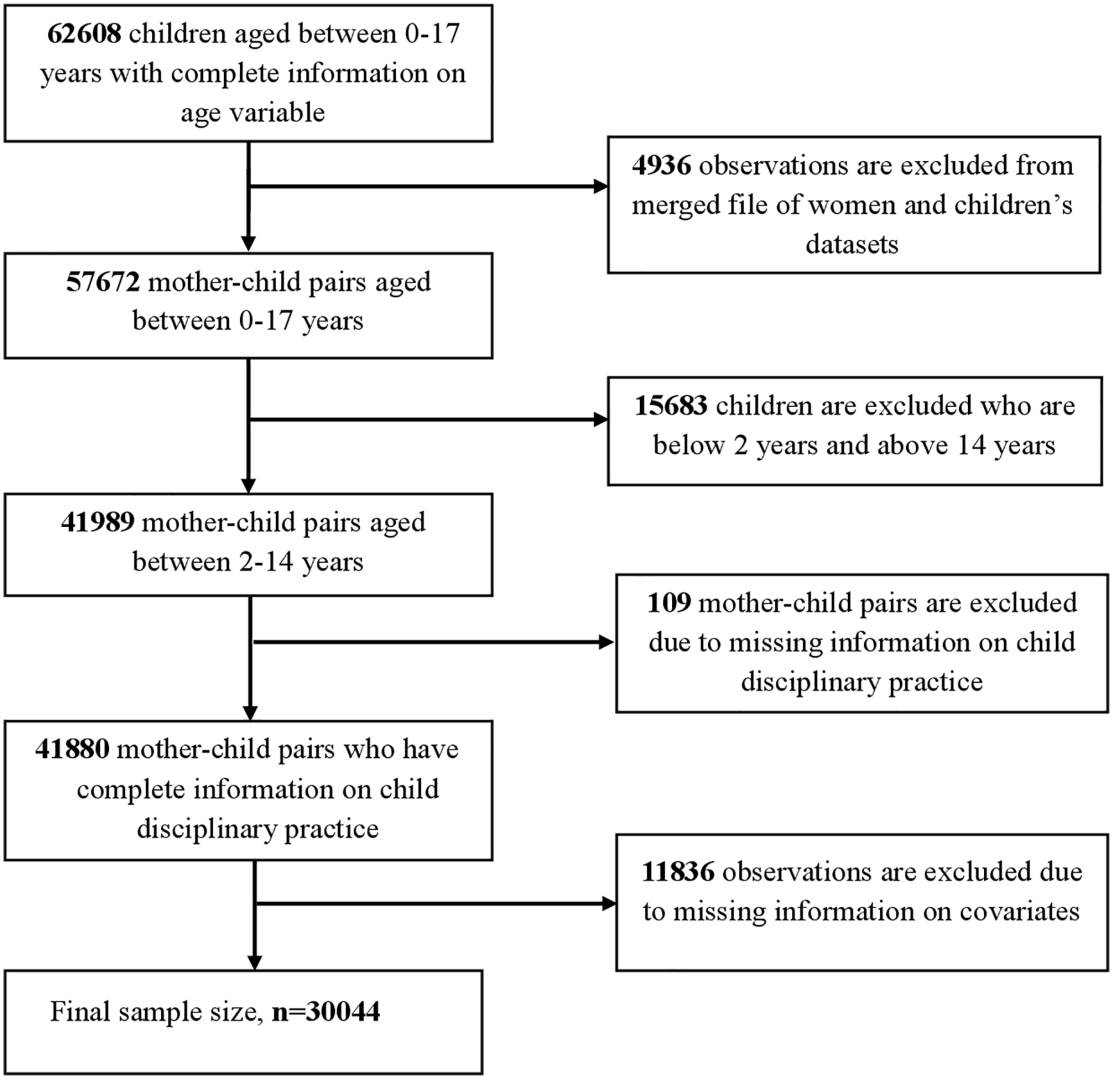
Flowchart of the target sample selecting scenarios.

The primary objective of this research was to investigate the relationship between physical disciplinary measures and a mother’s exposure to violence through the mediating role of the mother’s attitude towards IPV, disciplinary belief, and overall life satisfaction. The sample comprised children between the ages of 2 and 14 years, along with their mothers. Following the exclusion of incomplete cases and the alignment of maternal and child data, a total of 30044 mother-child pairs were included in the final analysis. **Fig 2** presents the flowchart depicting the scenarios for selecting the final sample size.

### 2.3. Ethical approval

The study made use of survey data from the 2019 Bangladesh MICS, in which all personal identifiers had been removed. The technical committee of the Government of Bangladesh, under the direction of the BBS, authorized the survey procedure, which contained precautions to mitigate any possible concerns. Verbal informed consent was obtained from all respondents. Participation was voluntary where confidentiality and anonymity were strictly maintained.

### 2.4. Data and variables

#### Outcome variable: Physical disciplinary practice.

The outcome variable of this study was the physical disciplinary practice at home. The Child Discipline Module included thorough details on disciplinary procedures, which was used to generate the response variable. Physical disciplinary practice, the outcome variable was computed from the reported responses of the mother or primary caregiver from six cases: shaking the child; spanking, hitting or slapped child on bottom with bare hand; hitting child on the bottom or elsewhere with belt, brush, stick etc.; hitting or slapping child on the face, head or ears; hitting or slapping child on the hand, arm or leg; and beating child up as hard as one could [6,15].

This study conceptualized physical disciplinary severity along two dimensions. The Moderate dimension included shaking a child; spanking, hitting, or slapping on the bottom with a bare hand; hitting on the bottom or elsewhere with a belt, brush, stick, or another object; and hitting or slapping the hand, arm, or leg. On the other hand, the severe dimension included hitting or slapping the face, head, or ears, and beating a child as hard as one could. Physical disciplinary practice was operationalized as a multidimensional composite index following the Alkire and Foster framework, with equal weights assigned to each dimension and these weights were further distributed equally among the indicators within each dimension [20,21]. Details about the dimensions, indicators and weights are displayed in **Table 1**.

**Table 1:** Dimensions, indicators, and weights of physical disciplinary practices towards children.

Dimension	Indicator	Weight (Indicator)	Weight (Dimension)
Moderate physical disciplinary practices	Shaking a child	0.125	0.5
	Spanking, hitting, or slapping a child on the bottom with a bare hand	0.125	
	Hitting a child on the bottom or elsewhere with a belt, brush, stick, or other object	0.125	
	Hitting or slapping a child on the hand, arm, or leg	0.125	
Severe physical disciplinary practices	Hitting or slapping a child on the face, head, or ears	0.25	0.5
	Beating a child as hard as one could	0.25	

A weighted arithmetic mean was calculated to obtain an individual physical disciplinary severity score. All the six indicators were dichotomized, with a value of 1 assigned when the respondent reported the disciplinary act and 0 when they did not. Mathematically, the score for individual i was formulated as given below.


$$Z_{i}=\left(\frac{\text{1}}{m}\sum_{j=\text{1}}^{m}\frac{\text{1}}{p_{j}}\sum_{k=\text{1}}^{p_{j}}y_{ijk}\right)$$

In the above equation $Z_{i}$ represents the physical disciplinary severity score for the $i^{th}$ (i=1,2,…,n) respondent; m is the number of dimensions (Moderate, Severe), $p_{j}$ is the number of indicators in the $j^{th}$ dimension (four indicators in moderate, two indicators in severe), $u_{ijk}$ is the value of the $k^{th}$ indicator in the $j^{th}$ dimension for the $i^{th}$ individual and n is the total number of respondents. For analyzing the extent of severity, $Z_{i}$ was grouped into three categories: no (for $Z_{i}=\text{0}$), moderate (for ${\text{0}<Z}_{i}<\text{0}.\text{5}$), and severe (for $Z_{i}\geq \text{0}.\text{5}$).

#### Main exogenous variable: Maternal exposure to violence.

The variable maternal exposure to violence, which characterizes an assault as a physical attack, was taken from the Victimization module of the MICS survey and utilized as the primary exposure of the current study. The exposure variable was categorized into two groups: unexposed (including the respondents who did not experience any type of physical attack) and exposed (encompassing the respondents reported as being physically attacked).

#### Mediator 1: Maternal attitude towards intimate partner violence (IPV).

The first mediator, maternal attitudes towards IPV, was selected from the Domestic Violence module. Respondents answered whether or not spousal abuse is justified in the following circumstances: going out without telling husband, neglecting the children, arguing with husband, refusing to have sex with husband, and burning the food. A composite score was created by aggregating the number of situations endorsed by the respondent, and finally a binary mediator variable—following the prior literature [22,23]—was derived to indicate two categories: “Does not justify IPV” for respondents who did not justify violence, and “Justifies IPV” for those who did [24–26].

#### Mediator 2: Maternal disciplinary belief.

Mothers or caregivers were questioned on whether they regarded physical punishment as a necessary measure for the appropriate upbringing, rearing, or education of a child. The binary variable maternal disciplinary beliefs, derived from the Child Discipline module, was employed as the mediating variable. Respondents who perceived physical punishment as a necessary measure of disciplining a child were assigned to the “supports physical punishment” category while their counterpart belonged to the “opposes physical punishment” group [27,28].

#### Mediator 3: Maternal life satisfaction.

Life satisfaction evaluation was measured among mothers/caregivers using a self-anchoring ladder scale ranging from 1 to 10, in which the top denoted the best possible life and the bottom the worst. The participants selected the rung that could best reflect their current life. Based on these responses, the maternal life satisfaction was categorized into three groups: high (8–10), moderate (4–7), and low (0–3).

#### Control variables.

Following the previous studies [28–33], sex of child (male and female), age of child (2–4, 5–9 and 10–14), child’s functional difficulty (yes and no), place of residents (urban and rural), maternal exposure to media (exposed and unexposed), wealth index (poor, middle and rich), mother’s education level (illiterate, primary, secondary, and higher) and mother’s age during first birth (≤18 years and >18 years) were considered as control variables in this study.

### 2.5. Statistical analyses

The data extracted from the MICS 2019 survey were prepared for analysis after excluding the missing or incomplete observations. Univariate analyses were performed to provide the frequency and percentage distributions of the selected variables. Subsequently, bivariate analyses examined the unadjusted associations between the outcome variable and each covariate, depicting the percentage distribution of responses within the categories of the corresponding covariate. The statistical significance of these associations was assessed using the chi-square test of independence [34]. The multivariate analyses relied on the ordinal logistic regression using the proportional odds assumptions under SEM framework [35]. The outcome variable was modeled considering the exposure, three mediator variables and all other control variables [36].

### 2.6. Structural equation modeling

The SEM approach, which is well suited for simultaneous estimation of multiple direct and indirect pathways, was employed in this study to examine the indirect link between maternal exposure and violence on the physical disciplinary practice through three potential mediators: maternal attitudes toward IPV, beliefs about child discipline, and overall life satisfaction. The mediation analysis was conducted using the product of coefficients method within an SEM framework [37–39]. Due to the binary nature of the first two mediators (maternal attitude towards IPV and disciplinary belief), binary logistic regression models were used as mediator models [40]. The mediator model for the maternal life satisfaction, an ordinal variable with three categories, relied on ordinal logistic regression model. The ordinal outcome motivated to use ordinal logistic regression under the SEM framework.

Let us assume that the physical disciplinary severity outcome $Y_{i}$ take C ordered categories for $i^{th}$ (i=1, 2, ⋯, n) individual and $E_{i}$ represents the responses on the exposure obtained from the $i^{th}$ individual, while $M_{\text{1}i}$ and $M_{\text{2}i}$ indicate the response on the first two mediators. Let also assume that the mediator 3, denoted by $M_{\text{3}i}$, has D ordered categories and the vector of control variables and corresponding regression coefficients are denoted respectively by $\begin{array}{c} X_{i}={\left(X_{i\text{1}},X_{i\text{2}},\cdots \;,X_{ip}\right)}^{T} \end{array}$ and $\begin{array}{c} \beta_{l}={\left(\beta_{\text{l1}},\beta_{\text{l2}},\cdots \;,\beta_{lp}\right)}^{T} \end{array}$, where l=1,2,3,4. Each ${\;\epsilon }_{i}$ indicates the random error term. The outcome and mediator models (I, II, and III) are formulated as given below.


$$\text{Outcome}\;\text{Model:}\;\text{log}\left(\frac{\text{Pr}(Y_{i}\leq c)}{\text{1}-\text{Pr}(Y_{i}\leq c)}\right)=\alpha_{\text{1}}-\lambda_{\text{1}}E_{i}-\lambda_{\text{2}}M_{\text{1}i}-\lambda_{\text{3}}M_{\text{2}i}-\;\lambda_{\text{4}}M_{\text{3}i}-{\;X}_{i}^{T\;\;}\beta_{\text{1}}+\;\;\epsilon_{i\text{1}}\;$$

$$\text{Mediator}\;\text{Model}\;\text{I:}\;\text{log}\left(\frac{\text{Pr}\;\left(M_{\text{1}i}=\text{1}\right)}{\;\text{1}-\text{Pr}\;\left(M_{\text{1}i}=\text{1}\right)}\right)=\alpha_{\text{2}}+\gamma_{\text{1}\;}E_{i}+{{\;X}_{i}^{\prime }\;\beta }_{\text{2}}+{\;\epsilon }_{i\text{2}}\;$$

$$\text{Mediator}\;\text{Model}\;\text{II:}\;\text{log}\left(\frac{\text{Pr}\;\left(M_{\text{2}i}=\text{1}\right)}{\;\text{1}-\text{Pr}\;\left(M_{\text{2}i}=\text{1}\right)}\right)=\alpha_{\text{3}}+\gamma_{\text{2}\;}E_{i}+{{\;X}_{i}^{\prime }\;\beta }_{\text{3}}+{\;\epsilon }_{i\text{3}}\;\;\;$$

$$\text{Mediator}\;\text{Model}\;\text{III:}\;\text{log}\left(\frac{\text{Pr}\left(M_{\text{3}i}\leq d\right)}{\text{1}-\text{Pr}\left(M_{\text{3}i}\leq d\right)}\right)=\alpha_{\text{4}}-\gamma_{\text{3}}E_{i}-X_{i}^{\prime }\beta_{\text{4}}+\epsilon_{i\text{4}}$$

In the above equations, each α stands for the intercept of the corresponding regression model. The λ’s stand for the direct associations of the exposure and mediators with the response variable while γ’s represent the direct associations between the exposure and three mediators. The product of coefficients approach is employed to estimate the mediating function of maternal attitude towards IPV, disciplinary belief and life satisfaction by multiplying the relevant path coefficients estimated through the maximum likelihood estimation approach [41]. Adjusted odds ratios (OR) were computed and demonstrated with 95% confidence interval (CI) and a 5% threshold of statistical significance was taken into consideration throughout the analysis. The bias-corrected standard errors were obtained using a bootstrapping technique, which does not rely on normality assumptions and accounts for sampling variability to ensure the validity of the estimates of indirect and total associations, providing a robust inference and stable mediation estimates [42]. All statistical analyses were conducted using Stata of version 17 and R programming of version 4.1.1.

## 3. Results

### 3.1. Exploratory analysis

**Table 2** summarizes the frequency and percentage distributions of all the selected variables. In the study sample, nearly half of the children under study (43.07%) experienced moderate physical disciplining while 22.23% were exposed to severe physical disciplining.

**Table 2:** Descriptive statistics of selected background characteristics by physical disciplinary practice along with *p*-value.

Characteristics (*n*)	Percentage (%)	Physical disciplinary practice			*p*-value
		No (34.7%)	Moderate (43.07%)	Severe (22.23%)	
**Maternal Exposure to Violence**					
Unexposed (28627)	95.28	35.39	43.11	21.50	<0.001
Exposed (1417)	4.72	20.61	42.27	37.12	
**Maternal Attitude towards IPV**					
Does not justify IPV (21444)	71.38	35.87	42.65	21.48	<0.001
Justifies IPV (8600)	28.62	31.74	44.13	24.13	
**Maternal Disciplinary Belief**					
Opposes Physical Punishment (19582)	65.18	40.86	42.29	16.84	<0.001
Supports Physical Punishment (10462)	34.82	23.13	44.53	32.34	
**Maternal life satisfaction**					
High (6161)	20.51	36.93	43.22	19.85	<0.001
Moderate (19441)	64.71	34.36	43.38	22.26	
Low (4442)	14.78	33.03	41.51	25.46	
**Sex of child**					
Male (15799)	52.59	32.20	43.34	24.45	<0.001
Female (14245)	47.41	37.44	42.77	19.78	
**Age of child (years)**					
2–4 (10432)	34.72	23.50	50.23	26.27	<0.001
5–9 (9322)	31.03	27.15	46.25	26.60	
10–14 (10290)	34.25	52.87	32.93	14.19	
**Child’s functional difficulty**					
No (28250)	94.03	34.67	43.62	21.71	<0.001
Yes (1794)	5.97	35.01	34.50	30.49	
**Place of residents**					
Urban (5769)	19.20	35.36	43.01	21.63	0.348
Rural (24275)	80.80	34.53	43.09	22.38	
**Maternal exposure to media**					
Unexposed (12154)	40.45	34.86	43.14	22.00	0.702
Exposed (17890)	59.59	34.57	43.03	22.40	
**Wealth index**					
Poor (9611)	31.99	32.60	43.72	23.68	<0.001
Middle (10109)	33.65	33.76	43.28	22.96	
Rich (10324)	34.36	37.54	42.27	20.19	
**Mother’s education level**					
Illiterate (5234)	17.42	39.68	38.67	21.65	<0.001
Primary (8073)	26.87	33.84	42.69	23.47	
Secondary (13333)	44.38	32.24	44.93	22.83	
Higher (3404)	11.33	38.63	43.48	17.89	
**Mother’s age during first birth**					
≤ 18 years (11915)	39.66	34.17	41.94	23.89	<0.001
> 18 years (18129)	60.34	35.05	43.81	21.15	

Only 4.72% of the mothers were exposed to physical attack while 28.62% of the mothers justified partner violence, and 34.82% supported the use of physical punishment in disciplining children. More than half of the mothers (64.71%) reported as being moderately satisfied in their life while 20.51% were considered to be happy with their life. The sample was nearly balanced by child sex, evenly distributed across the age groups of children, with most of the respondents residing in rural areas and mothers exhibiting varied maternal education levels.

Among the mothers reported violent exposure, 42.27% adopted moderate disciplinary practices while 37.12% used severe disciplinary practices, indicating a highly significant association (*p* < 0.001). A justificatory attitude toward IPV depicted a greater likelihood of physically disciplining children (with *p* < 0.001).

In the context of maternal disciplinary beliefs, more than half of the responses (44.53% moderate and 32.34% severe) on endorsing physical punishment also reported its actual use reflecting a significant association with a *p*-value of less than 0.001. Maternal life satisfaction reported that only 19.85% of the highly satisfied mothers adopted severe physical practices while this enforcement is higher for the mothers with low life-satisfaction (25.46%).

The bivariate analysis identified sex and age of the child, child’s functional difficulty, wealth index, mother’s education level and their age during first birth as significant control variables. Male children (43.34% moderate and 24.45% severe) were more frequently subjected to physical punishment than those of the females (42.77% moderate and 19.78% severe). The highest exposure to moderate disciplining (50.23%) was observed among children aged between 2 and 4 years while child disciplinary practices, both moderate and severe, were least employed on the children aged from 10 to 14 years. Respondents from both the middle and poor community were found to engage in child disciplinary practices at comparable frequencies. On the other hand, illiterate mothers showed minimal (38.67%) involved in moderate child disciplining while severe punishment methods were least (17.89%) applied by the higher educated respondents.

### 3.2. Outcome and mediator models

Results obtained from logistic regression models for the outcome and mediators are presented in **Table 3**. It is shown that mothers exposed to violence had an OR of 1.77 for implementing more harsh disciplinary actions on their children. This finding implies that victimized mothers had (1.77−1) ×100% = 77% higher odds of enacting more severe categories of physical disciplinary measures compared to those of the mothers with no such exposure. The difference between these two groups was found statistically significant (with *p* < 0.001). Mothers justifying spousal violence also showed 9% greater odds of applying more harsh physical discipline while 124% higher odds of the same was reported for the mothers who endorsed physical punishment compared to those of their counterparts. Table 3 also depicts that mothers with low and moderate life satisfaction were observed respectively with 15% and 9% higher odds of enacting more severe physical punishment compared to the highly satisfied mothers. All the three mediators illustrated significant association (with *p* < 0.001) with the outcome of interest, that is, physical disciplinary practice.

**Table 3:** Results of outcome and mediator models (I, II and III) displaying odds ratio (OR), *p*-values and 95% confidence intervals (CI).

Characteristics	Outcome model	Mediator Model I	Mediator Model II	Mediator Model III
	OR [95% CI]	OR [95% CI]	OR [95% CI]	OR [95% CI]
**Maternal exposure to violence**				
Unexposed (ref.)				
Exposed	1.77*** [1.60, 1.96]	1.14* [1.02, 1.28]	1.67*** [1.51, 1.87]	2.10***[1.89, 2.34]
**Maternal attitude towards IPV**				
Does not justify IPV (ref.)				
Justifies IPV	1.09*** [1.04, 1.15]			
**Maternal disciplinary belief**				
Opposes Physical Punishment (ref)				
Supports Physical Punishment	2.24*** [2.13, 2.34]			
**Maternal life satisfaction**				
High (ref)				
Moderate	1.09***[1.03, 1.15]			
Low	1.15***[1.06, 1.24]			
**Sex of child**				
Male (ref.)				
Female	0.78*** [0.75, 0.82]	0.94** [0.90, 0.99]	0.90*** [0.86, 0.95]	1.00 [0.96, 1.05]
**Age of child (years)**				
2–4 (ref.)				
5–9	0.88*** [0.83, 0.93]	0.97 [0.91, 1.03]	1.01 [0.95, 1.07]	1.04 [0.98, 1.10]
10–14	0.31*** [0.29, 0.33]	0.95 [0.89, 1.01]	0.85*** [0.80, 0.90]	0.97 [0.92, 1.03]
**Child’s functional difficulty**				
No (ref)				
Yes	1.23*** [1.12, 1.35]	1.02 [0.92, 1.14]	1.33*** [1.21, 1.47]	1.06 [0.96, 1.17]
**Place of residents**				
Urban (ref.)				
Rural	0.94 [0.89, 1.00]	0.98 [0.92, 1.06]	1.03 [0.97, 1.11]	0.89*** [0.84, 0.95]
**Maternal exposure to media**				
Unexposed (ref.)				
Exposed	1.12*** [1.07, 1.18]	0.90*** [0.84, 0.95]	0.98 [0.93, 1.04]	0.85*** [0.81, 0.92]
**Wealth index**				
Poor (ref.)				
Middle	1.01 [0.95, 1.07]	0.87*** [0.82, 0.93]	0.88*** [0.82, 0.93]	0.57*** [0.54, 0.62]
Rich	0.90** [0.84, 0.96]	0.62*** [0.57, 0.67]	0.78*** [0.73, 0.84]	0.31*** [0.29, 0.34]
**Mother’s education level**				
Illiterate (ref.)				
Primary	1.06 [0.99, 1.13]	0.83*** [0.79, 0.89]	1.03 [0.96, 1.11]	0.74*** [0.69, 0.79]
Secondary	1.02 [0.96, 1.08]	0.67*** [0.63, 0.71]	0.95 [0.89, 1.02]	0.46*** [0.43, 0.50]
Higher	0.79***[0.74, 0.89]	0.40*** [0.37, 0.45]	0.70*** [0.63, 0.78]	0.33*** [0.29, 0.35]
**Mother’s age during first birth**				
≤ 18 years (ref.)				
> 18 years	0.94** [0.90, 0.99]	0.93** [0.88, 0.98]	0.89*** [0.84, 0.93]	0.96 [0.92, 1.01]
**Threshold Values** (In Logit Scale)	**No | Moderate** **−**1.18***[−1.31, −1.04]	**Intercept:** 0.82***[0.72, 0.93]	**Intercept:** 0.96 [0.83, 1.10]	**High | Moderate:** −2.89***[−3.02, −2.77]
	**Moderate | Severe:** 0.91*** [0.78, 1.05]			**Moderate | Low:** 0.55***[0.43, 0.67]
**Log-likelihood**	−30010.41	−17522.45	−19186.65	−24888.11

Note. ref. indicates reference category, *p  <  0.05, **p  <  0.01, ***p  <  0.001.

Among significantly associated control variables, the sex of child depicted that the odds of enacting higher ranked physical punishment for female children were 0.78 times that of male children. In comparison with the children aged between 2–4 years, the odds of adopting more severe physical disciplinary measures were respectively 12% and 69% lower for children from the age groups of 5–9 and 10–14 years, respectively. The mothers exposed to media reported 12% higher odds of more harshly punishing their children physically compared to those of the unexposed mothers. The rich mothers were observed with 10% lower odds of adopting more strict disciplinary strategies compared to those from the poor community.

In contrast to illiterate mothers with no education, mothers with primary and secondary level of education illustrated respectively 6% and 2% higher odds of implementing harsher punitive measures towards children respectively while mothers with higher education exhibited 21% lower odds of the same.

The results of mediator models depicted the mothers with a history of physical abuse had significantly 14% higher odds (with *p* < 0.05) of endorsing positive attitudes towards IPV and 67% higher odds (with *p* < 0.001) of supporting strict disciplinary measures while 110% higher odds (with *p* < 0.001) of attaining a lower level of life satisfaction compared to those of the non-victimized mothers.

### 3.3. Mediation analysis

Results obtained from the mediation analysis considering structural equations after controlling for selected covariates are presented in **Table 4**. The results explained the direct and indirect relations of maternal exposure to violence with child physical disciplinary practices, mediated through maternal attitude towards IPV, disciplinary belief and life satisfaction, reflecting its overall association.

**Table 4:** Direct, indirect and total associations of maternal exposure to violence on physical disciplinary practices obtained from structural equation modelling.

Mediation	Characteristics	β (SE)	OR [95% CI]
**Direct Association**	**Maternal exposure to violence**		
	Unexposed (ref.)		
	Exposed	0.57 (0.05)	1.77***[1.60, 1.95]
**Indirect Association via maternal attitude towards IPV**	**Maternal exposure to violence**		
	Unexposed (ref.)		
	Exposed	0.01 (0.006)	1.02* [1.00, 1.04]
**Indirect Association via maternal disciplinary belief**	**Maternal exposure to violence**		
	Unexposed (ref.)		
	Exposed	0.41 (0.05)	1.51***[1.37, 1.66]
**Indirect Association via maternal life satisfaction**	**Maternal exposure to violence**		
	Unexposed (ref.)		
	Exposed	0.06 (0.02)	1.06*** [1.02, 1.10]
**Total Indirect Association**	**Maternal exposure to violence**		
	Unexposed (ref.)		
	Exposed	0.48 (0.05)	1.62***[1.47, 1.78]
**Total Association**	**Mother’s Exposure to Violence**		
	Unexposed (ref.)		
	Exposed	1.06 (0.07)	2.89***[2.52, 3.31]

Note. ref. indicates reference category, β indicates parameter estimates, SE indicates standard error, *p < 0.05, **p < 0.01, ***p < 0.001.

Maternal experience of physical violence demonstrated a significant direct association with 77% higher odds for practicing higher levels of physical disciplinary measures compared to those of their counterparts. The findings on the indirect association reveal that the mothers’ exposure to physical assault, being mediated through their attitude towards IPV, increased the odds of employing more severe physical punishment by 2% compared to those of the non-victimized mother. Although the estimated increase in the odds appears small, it is statistically significant (*p* < 0.05) and remains meaningful given the population-level implications of even modest shifts in harsh disciplinary practices. This result is found statistically significant with a *p*-value of less than 0.05. Furthermore, the mediating role of maternal victimization by violence through disciplinary belief illustrated 51% greater odds of adopting higher ranked physical disciplinary practices compared to their counterparts, which depicted a highly significant association (*p* < 0.001). Maternal life satisfaction mediating the association between mothers’ violence exposure and child physical discipline, resulted in 6% higher odds of more harshly punishing children compared to those of the unexposed mothers (*p* < 0.001). The total indirect association (via potential mediators) and total association showed that maternal exposure to violence significantly (*p* < 0.001) increased the odds of employing severe physical discipline toward children by 62% and 189%, respectively, relative to the non-victim counterparts.

To reexamine the potential heterogeneity, the regression models under SEM framework along with the bootstrapping, were conducted separately across the key demographic and socioeconomic subgroups determined as significant control variables in the outcome model of interest. All these subgroup-specific results are summarized in S1 Table.

## 4. Discussion

This study investigated the practice of physical discipline at home among children aged between two and fourteen years in Bangladesh, which was encountered with a considerably high prevalence (43.07% moderate and 22.23% severe). Comparable results have been reported in previous studies in countries such as Bangladesh, Vietnam, and Ghana, which reported the continued use of physical disciplining [29,43–45]. Despite the availability of substantial research evidence, these practices persist, largely sustained by cultural acceptance, and limited awareness about their adverse long-term consequences.

The main exogenous variable of this study, maternal exposure to physical attack, displayed higher likelihood of enacting physical punitive measures on children. This finding was consistent with a prior study conducted in Latin America and the Caribbean, where violent exposure in women was strongly associated with an increased likelihood of both verbal and physical abuse of children [46]. Shared risk factors can simultaneously increase the chances of both violent exposure and child physical discipline which serves as a potential reasoning for such finding [30,31].

Both the direct and indirect association with physical disciplinary practice were found significant through SEM. The current study revealed that exposure to violence depicted greater likelihood of coercive discipline through the mediator, attitude towards IPV. The women who justified spousal violence were more likely to use more severe punitive discipline with their children than those who disapproved such practices [26,47]. On the other hand, victimization of violence develops a positive attitude towards IPV [48]. Thus, the interplay between exposure to violence and attitudes justifying IPV, normalizes violent behavior within the household and perpetuates physical punishment of children [24,25].

The mediating role of maternal disciplinary belief, resulted in a much stronger indirect association between the violence exposure and disciplinary practice in contrast to the first mediator. This aligns with the previous research showing that parents who perceive physical punishment as an acceptable form of disciplining children are more inclined to applying it [27,49,50]. Thus, the endorsement of physical punishment normalizes its use as a disciplinary strategy.

Moreover, exposure to violence also encountered a significant indirect link through the mediator of maternal life satisfaction according to the findings of this study. This is also supported by the literature, which shows that higher happiness or life satisfaction of mother results in smaller odds for children experiencing disciplinary measures [16]. On the other hand, life satisfaction level usually decreases in women who are exposed to physical violence [51]. Thus, the interplay between maternal satisfaction of lower level and exposure to violence reinforces the use of physical punishment against children. Finally, the total association, including direct relation of maternal exposure to violence and its indirect relation through her violence-endorsing attitudes, beliefs and low satisfaction levels, corresponded to a strong link with much higher odds of using more stringent physical punishment. This demonstrates how maternal experience of violence in adulthood, strongly shapes parenting behavior by greatly increasing the likelihood of adopting physical punishment.

Among the control variables, it was found that boys were more frequently subjected to physical punishment than girls, influenced by societal expectations about appropriate behavior for each gender [28,32,52]. Boys are often seen as less obedient than girls, which encourages the use of physical discipline [33]. Older children tend to receive physical punishment less often according to the findings of this analysis, which confirmed the findings of previous research [53,54]. Younger children are more often subjected to physical discipline as they have less ability to assert themselves, misbehave more, and sometimes struggle to communicate with their parents. In contrast, older children, viewed as more mature and responsible, are often more disciplined through non-physical methods [55,56]. In this study, children from wealthier households experienced less physical punishment than those from poorer households. Greater economic stability reduces parental stress and frustration, provide access to supportive resources, allowing to meet family needs properly, thereby orienting towards positive parenting [57]. Mothers with higher secondary education showed lower involvement toward more severe physical disciplining, which is confirmed by previous studies [10,58]. The higher educated mothers being more aware of positive parenting strategies, rely less on aggression while more on communication and understanding to guide their children’s behavior [59].

## 5. Conclusion

The current study evaluated physical disciplinary practices from a number of questions on Child Discipline module using Bangladesh MICS 2019 data. Physical disciplinary practices are most common among mothers who have exposure to violence, normalize partner violence, and view physical punishment as an acceptable method of child discipline. Mothers’ exposure to physical violence acts as a key factor, while their attitudes towards IPV, disciplinary beliefs, and life satisfaction serve as critical mediators. The findings illustrated how tolerance of violence, endorsement of harsh physical discipline, and low levels of life satisfaction increase the likelihood of physically punishing children. Addressing these mediating pathways is essential for reducing harsh child disciplinary practices and reshaping attitudes toward IPV along with disciplinary beliefs.

### 5.1. Practical implications

The findings of this study suggested some policy implications to ensure a peaceful childhood environment. For example, promoting positive disciplinary beliefs can further help break intergenerational cycles of violence. Additionally, female education should be enhanced highlighting the protective role of women in society. A nurturing environment can be created for children by emphasizing child protective laws, addressing violence against women, changing the women’s attitude towards spousal abuse, and their disciplinary beliefs.

### 5.2. Strength and limitations

To the best of our knowledge, this study is the first to examine the association between mothers’ exposure to violence and adoption child physical disciplinary practices, while also exploring the mediating roles of maternal attitudes toward IPV, disciplinary beliefs, and overall life satisfaction level. The application of an SEM framework provided deeper insight into both the direct and indirect pathways linking maternal exposure to violence with disciplinary outcomes. Significant determinants were also evaluated selecting a number of control variables. In addition, physical discipline was modeled as an ordered outcome, with ordinal categories: none, moderate, and severe instead of a dichotomy. This retains meaningful gradations of harm and allows identification of potential determinants of more severe practices, providing more precise guidance for prevention. The study also highlights important policy implications, emphasizing the need for child protection laws, awareness campaigns on the harms of physical punishment, and educational initiatives for parents, particularly for mothers exposed to violence. Partnerships with NGOs and development agencies can further help shift societal attitudes and break the cycle of violence.

The limitation of this study is that it is cross-sectional in nature, assuming independence of observations and not accounting for potential clustering effects among children. Results are interpreted as associations rather than causal effects, given the absence for reverse causality. The question on whether the children coming from violence-prone homes will support and justify the violent disciplinary practice in their later life, is beyond the scope of this study. Although the study is informed by an intergenerational transmission framework, the data lack direct measures of children’s attitudes or later behaviors, and thus discussion of transmission is hypothesis-consistent rather than demonstrated. Testing causal pathways and intergenerational processes will require longitudinal designs and further studies can be designed to encompass these outlooks. On top of that, mothers may not report violence towards them and their children with enough transparency which may lead to under-reporting. It should also be noted that survey weights were not considered in the analysis since this study focused on identifying the direct and indirect association between the maternal violence and child disciplinary practice, rather than producing nationally representative estimates of specific indicators [60–62]. A further limitation is that the current study could not encompass variables like maternal stress, depression, anxiety, emotional abuse, sexual IPV, partner control, cultural norms, patriarchal structures, or religious beliefs since most of these variables were not directly available from the target survey. Additionally, some of these variables may be substantially under-reported in survey data, which could lead to incomplete measurement and limit the generalizability of our findings. Thus, this study focused exclusively only the physical forms of maternal violence, the most prevalent, observable, and reliably reported form of violence and child disciplinary practices. However, future studies can be designed to facilitate the full variation of maternal attitudes, encompassing psychological and emotional forms of violence to gain a more comprehensive understanding of the pathways linking maternal IPV to child discipline. Finally, the assessment of maternal life satisfaction represents a limitation of this study, as it is inherently difficult to measure and relies on self-reported scores that may not fully or accurately capture this latent construct.

## Supporting information

S1 Table Direct, Indirect and total associations between maternal violence and physical disciplinary practice based on different levels of covariates. (DOCX)
