# Supplementary material for: Intergenerational transmission of violence in Bangladesh: Mediated through maternal attitudes towards intimate partner violence, disciplinary beliefs, and life satisfaction
Source: PLoS One. 2026 Jan 30;21(1):e0341887. doi: 10.1371/journal.pone.0341887 (PMC12858018; doi:10.1371/journal.pone.0341887)
Supplement: S1 Table — (DOCX) [file pone.0341887.s001.docx]

**S1 Table: Direct, Indirect and total effects of maternal violence on physical disciplinary practice based on different levels of covariates**

| **Covariates** | **Estimated coefficient** | | |
| --- | --- | --- | --- |
|  | **Direct Association** | **Indirect Association** | **Total Association** |
| **Sex of child** | | | |
| Male | 0.65*** | 0.48** | 1.14** |
| Female | 0.53*** | 0.51*** | 1.04*** |
| **Age of child (years)** | | | |
| 2$-$4 | 0.60*** | 0.36*** | 0.96*** |
| 5$-$9 | 0.45*** | 0.56*** | 1.00*** |
| 10$-$14 | 0.68*** | 0.61*** | 1.30*** |
| **Child’s functional difficulty** | | | |
| No | 0.57*** | 0.49*** | 1.06*** |
| Yes | 0.65*** | 0.61** | 1.26*** |
| **Maternal exposure to media** | | | |
| Unexposed | 0.77*** | 0.47*** | 1.23*** |
| Exposed | 0.44*** | 0.51*** | 0.95*** |
| **Wealth index** | | | |
| Poor | 0.60*** | 0.46*** | 1.06*** |
| Middle | 0.56*** | 0.34*** | 0.90*** |
| Rich | 0.60*** | 0.81*** | 1.41*** |
| **Mother’s education level** | | | |
| Illiterate | 0.70*** | 0.49*** | 1.19*** |
| Primary | 0.60*** | 0.39*** | 0.98*** |
| Secondary | 0.50*** | 0.54*** | 1.04*** |
| Higher | 0.61* | 0.83** | 1.45*** |
| **Mother’s age during first birth** | | | |
| ≤18 years | 0.60*** | 0.49*** | 1.09*** |
| >18 years | 0.44*** | 0.51*** | 0.94*** |

Note: *p<0.05, **p<0.01, ***p<0.001
